# Endoscopic Management of Bouveret Syndrome With Electrohydraulic Lithotripsy

**DOI:** 10.14309/crj.0000000000001051

**Published:** 2023-05-10

**Authors:** Hunter Wang, Conner Blackmore, Mark Bonnichsen, George Ermerak, Milan Bassan

**Affiliations:** 1 Department of Gastroenterology and Hepatology, Liverpool Hospital, Sydney, Australia; 2 Department of Gastroenterology and Hepatology, Sydney Adventist Hosptial, Australia; 3 University of New South Wales, South Western Sydney Clinical School, Sydney, Australia

## CASE REPORTS

Bouveret syndrome is a rare form of gallstone ileus due to an impacted gallstone in the pylorus or duodenum after passage through a bilioenteric fistula. Traditional management is usually surgical with cholecystectomy (often open) and fistula repair and carries significant morbidity.^1,2^ Endoscopic management has been described in patients who are at high risk of surgery.^3^

We present 2 cases of Bouveret syndrome successfully managed endoscopically (Videos 1 and 2).

**Video 1:** Case 1.

**Video 2:** Case 2.

### Case 1

**Figure 1.**
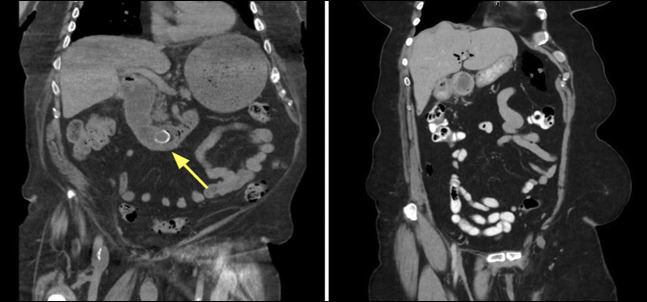
(A) Case 1: CT scan demonstrating a 35 mm gallstone in the third part of the duodenum with upstream dilatation (yellow arrow). (B) Case 2: CT scan demonstrating large ectopic gallstone, pneumobilia, and cholecystoduodenal fistula. CT, computed tomography.

A 96-year-old man from a nursing home presented with a 10-day history of abdominal pain, nausea, and vomiting. Computed tomography scan demonstrated gastric outlet obstruction with a 35 mm gallstone in the duodenum and a cholecystoduodenal fistula (Figure 1).

### Case 2

A 76-year-old woman presented with severe epigastric pain and a history of cholelithiasis. Computed tomography scan demonstrated a 29 mm gallstone in the first part of the duodenum with associated pneumobilia and a cholecystoduodenal fistula (Figure 1).

**Figure 2.**
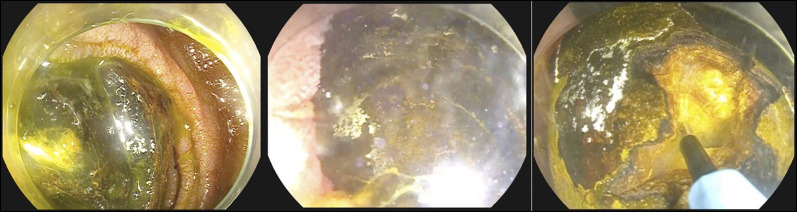
(A) Impacted gallstone in the third part of the duodenum. (B) Fragmentation of the gallstone with electrohydraulic lithotripsy. (C) Partially fragmented gallstone.

**Figure 3.**
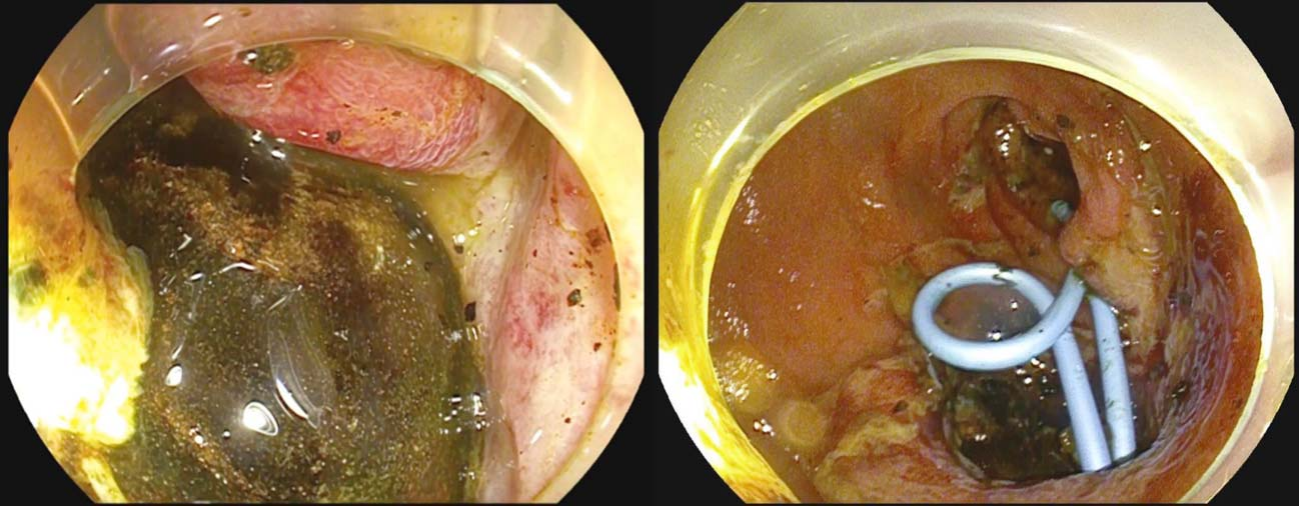
(A) Cholecystoduodenal fistula at the 4 o'clock position with pigtail stents in situ. (B) Impacted cholelithiasis at cholecystoduodenal fistula.

In both cases, the decision was made for initial endoscopic therapy because of unacceptable acute surgical risk. Electrohydraulic lithotripsy (EHL) using a standard biliary EHL probe (Autolith; Boston Scientific, Natick, MA) through a straight endoscopic retrograde cholangiopancreatography catheter with a gastroscope was performed. The stone was immersed in sterile saline to allow conduction of the EHL pulse (Figure 2) with power setting high and 30 pulses per activation. Complete fragmentation of the obstructing gallstone was successful in both cases, and stone fragments were retrieved. In case 1, double pigtail plastic stents were inserted across the cholecystoduodenal fistula allowing long-term gallbladder drainage (Figure 3). An additional treatment session was required to fragment a residual large stone within the gallbladder (Figure 3) to prevent further gallstone ileus. Both cases recovered without complication. Because case 1 was not a surgical candidate, his stents will remain permanently in situ. Case 2 had outpatient surgical review and awaits cholecystectomy. Both patients have had no further episodes of obstruction or sepsis.

This technique was first attempted by Morial et al4 with a recent systematic review by Ong et al5 describing the success rate of EHL in combination with another treatment modality such as mechanical lithotripsy or extracorporeal shock wave lithotripsy to be between 45%–100%. Although case series numbers are too small to guide selection criteria, favourable factors for endoscopic success include gallstone <4cm, impacted gallstone in proximal half of duodenum and multimodal therapy. Intraluminal EHL offers a minimally invasive therapeutic option for Bouveret syndrome and should be considered in patients who are poor operative candidates.

## DISCLOSURES

Author contributions: H. Wang, M. Bonnichsen, and C. Blackmore: writing—original draft and writing—review and editing. G. Ermerak and M. Bassan: conceptualization and writing—review and editing. All authors have read and approved the final version. C. Blackmore is the article guarantor.

Financial disclosure: None to report.

Informed consent was obtained for this case report.
